# Systemic intracellular analysis for balancing complex biosynthesis in a transcriptionally deregulated *Escherichia coli* l‐Methionine producer

**DOI:** 10.1111/1751-7915.14433

**Published:** 2024-03-25

**Authors:** Claudia Harting, Attila Teleki, Marius Braakmann, Frank Jankowitsch, Ralf Takors

**Affiliations:** ^1^ University of Stuttgart Stuttgart Germany; ^2^ Evonik Operations GmbH Halle‐Künsebeck Germany

## Abstract

l‐Methionine (l‐Met) has gained remarkable interest due to its multifaceted and versatile applications in the fields of nutrition, pharmaceuticals and clinical practice. In this study, the fluxes of the challenging l‐Met biosynthesis in the producer strain *Escherichia coli* (*E. coli*) DM2853 were fine‐tuned to enable improved l‐Met production. The potential bottlenecks identified in sulfur assimilation and l‐Met synthesis downstream of *O*‐succinyl‐l‐homoserine (OSHS) were addressed by overexpressing glutaredoxin 1 (*grxA*), thiosulfate sulfurtransferase (*pspE*) and *O*‐succinylhomoserine lyase (*metB*). Although deemed as a straightforward target for improving glucose‐to‐Met conversion, the yields remained at approximately 12%–13% (g/g). Instead, intracellular l‐Met pools increased by up to four‐fold with accelerated kinetics. Overexpression of the Met exporter *ygaZH* may serve as a proper valve for releasing the rising internal Met pressure. Interestingly, the export kinetics revealed maximum saturated export rates already at low growth rates. This scenario is particularly advantageous for large‐scale fermentation when product formation is ideally uncoupled from biomass formation to achieve maximum performance within the technical limits of large‐scale bioreactors.

## Graphical abstract

Metabolomic analysis revealed potential bottlenecks in the complex methionine biosynthesis of an *E. coli* producer strain. A genetic engineering strategy was designed to overcome these bottlenecks.

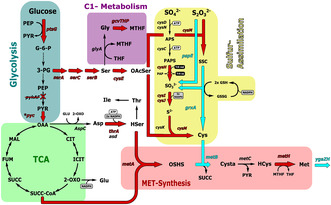

## INTRODUCTION

l‐Methionine (l‐Met) is a sulfur‐containing proteinogenic amino acid (Brosnan & Brosnan, 2006). Whereas most plants, fungi and bacteria can synthesize l‐Met, in animals (including humans), l‐Met is an essential amino acid (Rose, 1938; Willke, 2014). l‐Met deficiency has been associated with various diseases such as toxaemia, muscle paralysis, depression, schizophrenia, hair loss and growth disorders (Kumar & Gomes, 2005; Rose, 1938). l‐Met is used to treat liver dysfunction, allergies and rheumatic fevers (Dischert et al., 2013; Willke, 2014). The industrial‐scale application of Met has been used as an animal feed additive. This is due to the observation that l‐Met is the first and third most limiting amino acid in poultry and piglet feeds, respectively (Jankowski et al., 2014).

A major amount of Met is chemically synthesized (Lüssling et al., 1981), and this results in a racemic mixture of DL‐Met. To obtain pure l‐Met, additional processing steps such as enzymatic conversion, extraction and chromatography are necessary (Wöltinger et al., 2005). Alternatively, the l‐Met precursors *O*‐succinyl‐l‐homoserine (OSHS) or *O*‐acetyl‐l‐homoserine (OAHS) are produced through fermentation and then enzymatically converted to pure l‐Met (Hong et al., 2012). However, this process is only suitable for special applications in medicine and pharmacies. Therefore, a fermentation process using natural resources to produce pure l‐Met provides an alternative approach. The complex biosynthesis pathway of l‐Met has prevented the development of a commercially competitive fermentation process (Willke, 2014).

**FIGURE 1:**
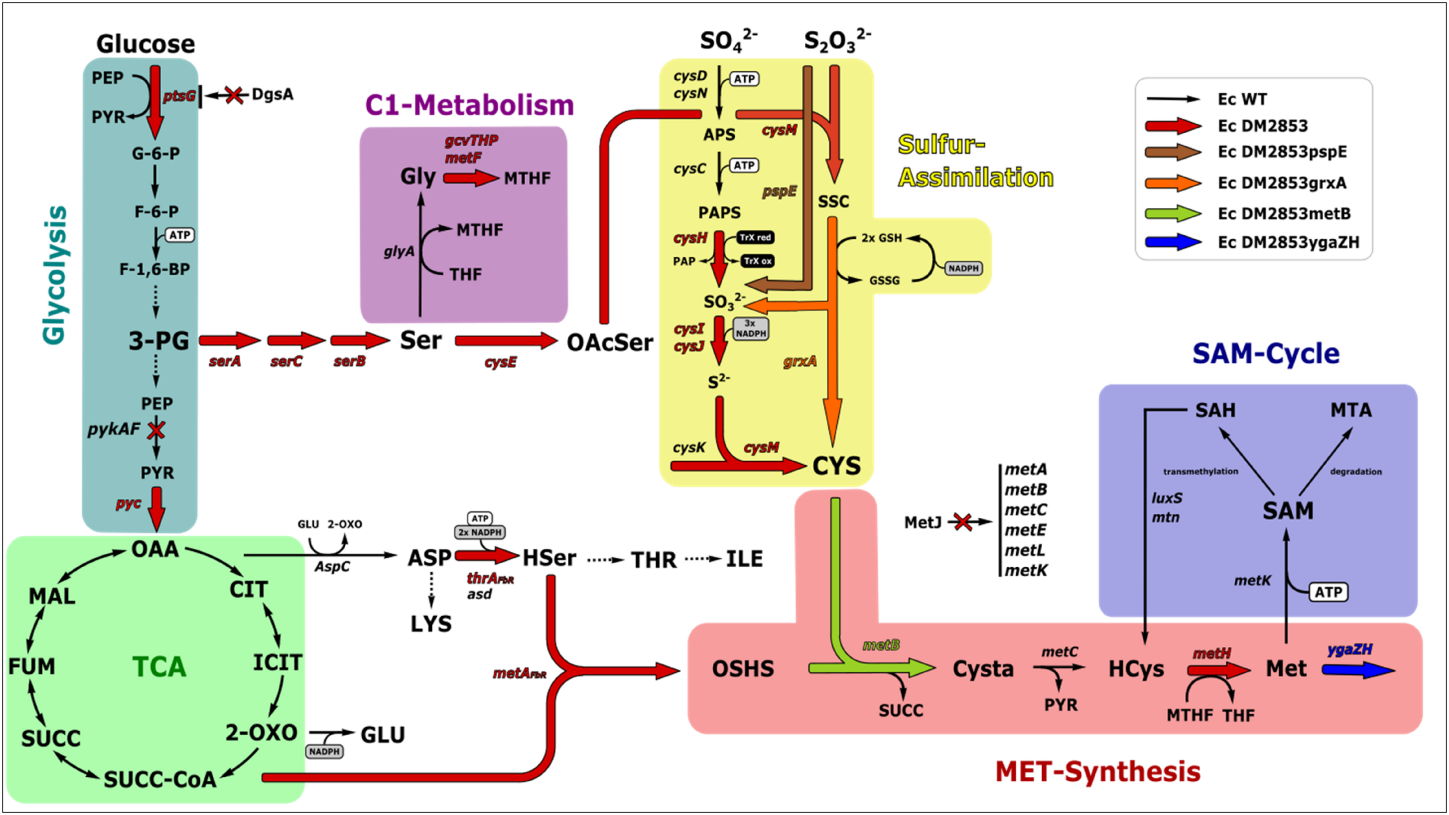
Biosynthesis pathway of l‐methionine in *E.coli*. Black arrows depict wild type enzymatic reaction. Thick arrows depict overexpressed enzymatic reactions of the engineered *E. coli* strains. Dotted arrow indicates more than one enzymatic reaction. Red cross indicates knockout. 2‐OXO, 2‑oxoglutarate; 3‑PG, 3‑phosphoglycerate; APS, adenosine phosphosulfate; ASP, l‐aspartate; CIT, citrate; Cys, l‑cysteine; Cysta, l‐cystathionine; F‐1,6‐BP, fructose‐1,6‐bisphosphate; F‐6‐P, fructose‐6‐phosphate; FUM, fumarate; G‐6‐P, glucose‐6‐phosphate; GLU, l‐glutamate; Gly, l‐glycine; GSH, reduced glutathione; GSSG, oxidized glutathione; HCys, l‐homocysteine; HSer, l‐homoserine; ICIT, Isocitrate; ILE, l‐isoleucine; LYS, l‐lysine; MAL, malate; Met, l‑methionine; MTA, methylthioadenosine; MTHF, 5‐methyltetrahydrofolate; OAA, oxaloacetate; OAcSer, O‐acetyl‐l‐serine; OSHS, O‑succinyl‐l‐homoserine; PAPS, phosphoadenosine phosphosulfate; PEP, phosphoenolpyruvate; PYR, pyruvate; S^2‐^, sulfide; S_2_O_3_^2‐^, thiosulfate; SAH, S‐adenosyl‐l‐homocysteine; SAM, *S*‐adenosyl‐l‐methionine; Ser, l‐serine; SO_3_^2‐^, sulfite; SO_4_^2‐^, sulfate; SSC, S‐sulfo‐l‐cysteine; SUCC, succinate; SUCC‐CoA, succinyl‐coenzyme A; THF, tetrahydrofolate; THR, L‐threonine; ptsG encoding member of glucose‐specific phosphotransferase system, dgsA encoding transcriptional regulator of carbohydrate metabolism, pykAF encoding Pyruvate kinase I+II, pyc encoding pyruvate carboxylase, aspC encoding aspartate aminotransferase, thrA encoding aspartate kinase I, asd encoding aspartate‐semialdehyde dehydrogenase, metA encoding homoserine O‐succinyltransferase, metB encoding O‐succinylhomoserine lyase, metC encoding cystathionine β‐lyase, metH encoding cobalamin‐dependent methionine synthase, ygaZH encoding branched chain amino acid exporter, metK encoding methionine adenosyltransferase, metE encoding cobalamin‐independent methionine synthase, metL encoding bifunctional aspartate kinase II/homoserine dehydrogenase II, metJ encoding transcriptional methionine repressor, serA encoding phosphoglycerate dehydrogenase, serC encoding phosphoserine aminotransferase, serB encoding phosphoserine phosphatase, glyA encoding serine hydroxymethyltransferase, gcvT encoding aminomethyltransferase, gcvH encoding glycine cleavage system H protein, gcvP encoding glycine decarboxylase, metF encoding 5,10‐methylenetetrahydrofolate reductase, cysE encoding serine acetyltransferase, cysK encoding cysteine synthase A, cysM encoding cysteine synthase B, cysDN encoding sulfate adenylyltransferase, cysC encoding adenylyl‐sulfate kinase, cysH encoding phosphoadenosine phosphosulfate reductase, cysIJ encoding sulfite reductase, pspE encoding thiosulfate sulfurtransferase, grxA encoding reduced glutaredoxin 1.

The de novo synthesis of l‐Met is very energy demanding, requiring 7 mol of ATP as an energy equivalent and 8 mol of NADPH as an anabolic reducing agent per mol of l‐Met in *Escherichia coli* (*E. coli*) (Kaleta et al., 2013). To avoid wasteful overproduction, microorganisms synthesize only the amounts of l‐Met necessary for growth (Kumar & Gomes, 2005). Due to the intricate nature of l‐Met biosynthesis (Figure 1) that involves multiple branched pathways, finely tuned fluxes are required to ensure efficient production. Cysteine (Cys) branches from glycolysis via the synthesis of serine (Ser) and *O*‐acetyl‐l‐serine (OAcSer) (Hindson & Shaw, 2003; Kuznetsova et al., 2006). Sulfur required for Cys synthesis is acquired via reductive sulfur assimilation (Nakatani et al., 2012). C1 metabolism that branches off from Ser produces the methylation substrate 5‐methyltetrahydrofolate (MTHF) that is required in the final synthesis step of l‐Met (Okamura‐Ikeda et al., 1993; Schirch & Szebenyi, 2005). The carbon skeleton of l‐Met is derived from aspartate (Asp) that is formed from the TCA. Incorporation of sulfur into the carbon backbone of l‐Met occurs via the reaction of OSHS with Cys to form cystathionine (Cysta), and this is mediated by *O*‐succinylhomoserine lyase (encoded by *metB*) (Holbrook et al., 1990). Cysta is then converted to homocysteine (HCys) (Laber et al., 1996). Finally, methionine synthase (encoded by *metH*) methylates HCys with MTHF to form l‐Met (Whitfield et al., 1970). Furthermore, l‐Met synthesis is tightly regulated at multiple levels. With the exception of MetH, each enzyme in the l‐Met synthesis pathway undergoes transcriptional repression and/or allosteric inhibition. First and foremost the transcriptional methionine repressor MetJ acts on each gene along l‐Met synthesis, including aspartate kinase/homoserine dehydrogenase II encoded by *metL*, homoserine O‐succinyltransferase encoded by *metA*, *metB*, cystathionine β‐lyase encoded by *metC* and homocysteine transmethylase encoded by *metE*. Additionally, l‐Met itself regulates MetL, MetA and aspartate‐semialdehyde dehydrogenase that is encoded by *asd* (Kumar & Gomes, 2005). Transcriptional activation is mediated by the transcription factor MetR that regulates the expression of *metE* and *metH* (Weissbach & Brot, 1991).

In recent years, intensive strain development has been conducted to optimize this complex biosynthetic pathway for l‐Met production. Overexpression of phosphoserine phosphatase (encoded by *serB*), phosphoserine aminotransferase (encoded by *serC*) and serine acetyltransferase (encoded by *cysE*) have proven to be beneficial for optimizing the supply of the precursors Ser and OAcSer (Huang et al., 2018). C1 metabolism has also been the subject of strain development experiments. Overexpression of 5,10‐methylenetetrahydrofolate reductase (encoded by *metF*) increased l‐Met titer (Shen et al., 2023). In contrast, overexpression of serine hydroxymethyltransferase (encoded by *glyA*) did not lead to improved l‐Met production but instead reversed the enzymatic reaction, finally accumulating Ser (Shen et al., 2023; Tang, Du, et al., 2020). For sulfur assimilation, the upregulation of the thiosulfate/sulfate uptake system (encoded by *cysPUWA*), cysteine synthase A (encoded by *cysK*) and cysteine synthase B (encoded by *cysM*) is beneficial for l‐Met synthesis (Huang et al., 2018). *MetA* is an important node in l‐Met biosynthesis. Research has been conducted to identify effective feedback‐resistant mutants of *metA* that increase l‐Met titer after overexpression (Huang et al., 2017; Tang, Chen, et al., 2020; Tang, Du, et al., 2020). Regarding transcriptional regulation deleting the transcriptional repressor *metJ* has proven to be effective as well as overexpressing the l‐Met exporter (encoded by *yjeH*) (Huang et al., 2017; Liu et al., 2015).

In this study, the *E. coli* strain DM2853 was used to investigate the complex regulatory network of l‐Met production. DM2853 combines many of the aforementioned modifications and contains additional genetic changes. An overview of these genetic changes is presented in Figure 1. Within glycolysis, the strain contains alterations that include an additional copy of *ptsG*, a member of the glucose‐specific phosphotransferase system, with the trc promoter (a hybrid of the lac and trp promoters) on an episomal vector. Global transcriptional regulator of carbohydrate metabolism (encoded by *dgsA*) and pyruvate kinases I and II (encoded by *pykAF*) are deleted. Heterologous overexpression of pyruvate carboxylase (encoded by *pyc*) promotes the synthesis of oxaloacetate. The complete serine pathway leading to OAcSer that comprises phosphoglycerate dehydrogenase (encoded by *serA*), *serC*, *serB* and *cysE* is overexpressed. Within C1‐metabolism, the glycine cleavage system (encoded by *gcvTHP*) and *metF* are overexpressed. Within the sulfur assimilation pathway, the sulfate and thiosulfate ABC transporter (encoded by *cysPUWA*), phosphoadenosine phosphosulfate (PAPS) reductase (encoded by *cysH*), sulfite reductase (encoded by *cysIJ*) and *cysM* are overexpressed. Multiple copies of a mutated version of bifunctional aspartokinase/homoserine dehydrogenase 1 (encoded by *thrA*) that is feedback‐resistant to threonine are inserted into the genome. Similarly, there are multiple copies of *metA* Q64E integrated into the genome that is feedback resistant against methionine and S‐adenosyl‐l‐methionine (SAM) (Tang, Chen, et al., 2020). The transcriptional methionine repressor *metJ* is deleted, as it represses almost every enzyme involved in methionine synthesis (MetA, B, C, E, L and K). The final step in methionine synthesis is catalysed by *metH* that is overexpressed by the *trc* promoter. Therefore, our study aimed to investigate the potential bottlenecks and their correlations. It will be demonstrated that sophisticated metabolic profiling discloses potential shortcomings in sulfur assimilation and precursor supply. However, experimental observations have identified additional regulatory interactions that remain to be elucidated. Furthermore, amplifying export capacity may provide a straightforward target that can offer unexpected benefits to the fermentation operation mode.

## EXPERIMENTAL PROCEDURES

### Cultivation, seed train, medium

Unless otherwise stated, *E. coli* DM2853 (Dischert & Figge, 2013) and all its derivatives were cultivated at 37°C and 130 rpm (Infors HT; Infors AG, Bottmingen, Switzerland). Baffled shake flasks were filled to 10% capacity. To create a master cell bank (MCB), cells were cultivated in LB medium (10 g/L tryptone, 5 g/L yeast extract, 10 g/L NaCl) for 24 h. To ensure equal glycerol seed stocks that serve as a working cell bank (WCB), the following procedure was performed. Cells from the MCB were incubated on LB agar plates for 24 h, and this was followed by 24 h‐lasting cultivation in culture tubes with 90% minimal medium and 10% LB medium. Then, baffled shake flasks with 100% minimal medium were inoculated at an OD of 0.2, and WCB samples were finally frozen at OD 10. Experimental studies began with the preculture (baffled shake flask, 100% minimal medium) that was inoculated with 2 mL and cultured for 10–14 h. Then, the main culture (baffled shake flask, 100% minimal medium) was inoculated at an OD of 0.2 and cultivated at 200 rpm. Minimal medium consisted of: K_2_HPO_4_ (8 g/L); Na_2_HPO_4_ (2 g/L); (NH_4_)_2_HPO_4_ (8 g/L); NH_4_Cl (0.13 g/L); (NH_4_)_2_S_2_O_3_ (5.6 g/L); MgSO_4_·7H_2_O (1 g/L); CaCl_2_·2H_2_O (0.04 g/L); C_12_H_17_N_4_OS·HCl (0.01 g/L); C_62_H_88_CoN_13_O_14_P (0.01 g/L); C_6_H_12_O_6_ (15 g/L); C_6_H_8_O_7_·H_2_O (6 g/L); C_7_H_15_NO_4_S (15 g/L); ZnSO_4_·7H_2_O (0.004 g/L); CuCl_2_·2H_2_O (0.002 g/L); MnSO_4_·H_2_O (0.02 g/L); CoCl_2_·6H_2_O (0.008 g/L); H_3_BO_3_ (0.001 g/L); FeSO_4_·7H_2_O (0.04 g/L); C_9_H_18_O_5_S (0.0024 g/L) (pH 6.8).

### Construction of *E. coli* DM2853 overexpressing target genes

Standardized cloning procedures such as PCR and DNA restriction were performed according to Sambrook and Russell (2001). Plasmids were isolated from 100 mL liquid cultures using the E.Z.N.A.® Plasmid Midi Kit (Omega Bio‐tek, Inc., Norcross, USA) following the manufacturer's instructions. The genomic DNA of *E. coli* MG1655 was isolated using the DNeasy Blood & Tissue Kit (Qiagen, Hilden, Germany) according to the manufacturer's instructions. This DNA was used to amplify the target genes *metB*, *grxA* and *pspE* via PCR with primers ch225/ch226, ch207/ch208 and ch217/ch218, respectively. PCR products were purified with NucleoSpin® Gel and a PCR Clean‐up Kit (Macherey‐Nagel GmbH & Co. KG, Düren, Germany) according to the manufacturer's instructions. Complementary regions of 30 bp to the plasmid backbone were added to the target genes via PCR using the following primers: ch247/ch248 (*metB*); ch237/ch238 (*grxA*); ch241/ch242 (*pspE*).

The non‐codon‐optimized sequence of *ygaZH* originates from *Citrobacter koseri* (Figge et al., 2016) (listed in Supplementary Information) and was synthetically synthesized (Thermo Fisher Scientific, Waltham, MA, USA). Primers ch352/ch353 were used to amplify *ygaZH*. After purification, complementary regions of 30 bp to the plasmid backbone were added to *ygaZH* by PCR using the primers ch354/ch355 (PM1‐12) and ch355/ch356 (PM1‐93). The plasmid backbone containing the promoter PM1‐93 was derived from pJOE5304 P93 *metK*. It was digested with BamHI and HindIII and amplified with ch25/ch231. The plasmid backbone containing the promoter PM1‐12 was derived from pJOE5304 P12 *metB*. It was linearized with AhdI and amplified using primers ch25/ch296. Cloning was performed using Gibson Assembly (Gibson et al., 2009). The Gibson reaction mix was desalted with a membrane filter (MF‐Millipor™ 0,025 μm, Merck KGaA, Darmstadt, Germany) and electroporated into competent *E. coli* DH5α. After sequencing, the plasmids were electroporated into competent *E. coli* DM2853 cells.

### Extracellular analysis

Biomass formation was monitored by measuring the OD_600_ or dry cell weight (DCW) (g/L) of the samples. The correlation factor of DCW (OD_600_ × 0.26) was identified through independent measurements.

To determine the glucose and amino acid concentrations in the supernatant, 1 mL of the culture was harvested by centrifugation (12,100*g*, 10 min, rt), and the cell‐free supernatant was analysed. Glucose concentration was quantified using enzyme kits (r‐biopharm AG, Darmstadt, Germany).

The ratio of intracellular versus extracellular volume is a time‐variant value that is valid for the conditions at 22 h. The calculation included the extracellular volume *V*_extra_ = 40 mL, DCW = 2415 mg/mL and the intracellular volume of *E. coli V*_Ec_ = 0.0023 mL/mg (Bennett et al., 2008).

$$\mathrm{DCW}\left[\mathrm{mg}\right]=\mathrm{DCW}*V_{\text{extra}}=\frac{2415\mathrm{mg}}{\mathrm{ml}}*40\;\mathrm{mL}=96.6\;\mathrm{mg},$$

$$V_{\text{intra}}=\mathrm{DCW}*V_{\mathrm{Ec}}=96.6\;\mathrm{mg}*\frac{0.0023\;\mathrm{mL}}{\mathrm{mg}}=0.222\;\mathrm{mL},$$

$$\frac{V_{\text{extra}}}{V_{\text{intra}}}=\frac{40\;\mathrm{mL}}{0.222\;\mathrm{mL}}=180.$$

### qPCR

RNA was isolated using the Quick RNase Mini Kit (Zymo Research, Irvine, CA, USA) according to the manufacturer's instructions. Isolated RNA was treated with Turbo DNAse (Thermo Fisher Scientific, Waltham, MA, USA) and concentrated using the RNA Clean and Concentrator 5 Kit (Zymo Research, Irvine, CA, USA). For complementary DNA (cDNA) synthesis, 1 μg of isolated RNA was treated with Reverse Transcriptase Superscript IV (Thermo Fisher Scientific, Waltham, USA) according to the manufacturer's instruction using random hexamers (NEB, Ipswich, USA). After RT‐PCR, 1 μL of RNaseH (NEB, Ipswich, USA) was added, and the samples were incubated at 37°C for 20 min to digest initial RNA. For quantitative PCR (qPCR), the cDNA samples were diluted with ddH2O by a factor of six. qPCR master mix contained 10 μL of 2× qPCR S'Green Blue Mix (Biozym Scientific GmbH, Hessisch Oldendorf, Germany), 0.8 μL of forward primer, 0.8 μL of reverse primer and 6.4 μL of ddH_2_O per reaction. The primers that were used are listed in Table 1. Samples were measured in technical triplicates. Controls included non‐RT and non‐template controls. A three‐step 1:10 dilution series of pooled cDNA was prepared to determine amplification efficiency. qPCR was conducted on a qTower^3^ (Analytik Jena, Jena, Germany) under the conditions: 95°C 2 min; 40 cycles of 95°C for 5 s, 62°C for 30 s; a final ramp from 65°C to 95°C (0.5°C steps every 5 s). The relative expression of the target genes was standardized to the expression of *cysG* (Zhou et al., 2011), and relative quantification was performed according to Pfaffl (2001).

**TABLE 1:** Bacterial strains, plasmids and oligonucleotides.

Strain/plasmid/primer	Strain information/sequence 5′ → 3′	Reference/purpose
*E. coli* DH5α λ *pir*	Cloning strain	Michalowski et al. (2017)
*E. coli* MG1655	Wild‐type strain	Michalowski et al. (2017)
*E. coli* DM2853	*E. coli* MG1655 metA*11 P*trc*‐*metH* P*trc*F‐*cysPUWAM* P*trc*F‐*cysJIH* P*trc*09‐*gcvTHP* P*trc*36‐ARNmst17‐*metF* P*trc*07‐*serB* Δ*metJ* Δ*pykF* Δ*pykA* Δ*purU* Δ*yncA* Δ*malS*::TT*adc*‐*CI857*‐PlambdaR*(−35)‐thrA*1‐*cysE* Δ*pgaABCD*::TT02‐TT*adc*‐PlambdaR*(−35)‐RBS01‐thrA*1‐*cysE*‐P*gapA*‐metA*11 Δ*uxaCA*::TT07‐TT*adc*‐PlambdaR*(−35)‐RBS01‐thrA*1‐*cysE*‐P*gapA*‐metA*11 Δ*CP4‐6*::TT02‐TT*adc*‐PlambdaR*(−35)‐RBS01‐thrA*1‐*cysE*‐P*gapA*‐metA*11 Δ*wcaM*:: TT02‐TT*adc*‐PlambdaR*(−35)‐RBS01‐thrA*1‐*cysE*‐P*gapA*‐metA*11 Δ*treBC*::TT02‐*serA‐serC* Δ*melB*::RN/P*trc*01/ARN01/RBS01*2‐*pycre*‐TT07 Δ*purU*::RN/PL1*1/RBS01*2‐*pycre*‐TT07 Δ*yjbI*::RN/P*trc*01/RBS01‐*gcvTHP*‐TT07 Δ*dgsA*::Km (pCC1BACVB01‐P*lacI*q‐*LacI*‐TT02‐P*trc*01/OP01/RBS01*2‐*ptsG*‐TT07‐P*trc*30/RBS01‐*serC*‐TT07*2‐P*trc*30/RBS01‐*serA*‐TT*adcca*)	Strain 17 in Dischert and Figge (2013)
*E. coli* DM2853 pJOE5304	DM2853 with pJOE5304 (empty vector)	This study
*E. coli* DM2853 *metB*	DM2853 with pJOE5304 *metB*	This study
*E. coli* DM2853 *grxA*	DM2853 with pJOE5304 *grxA*	This study
*E. coli* DM2853 *pspE*	DM2853 with pJOE5304 *pspE*	This study
*E. coli* DM2853 P93 *ygaZH*	DM2853 with pJOE5304 P93 *ygaZH*	This study
*E. coli* DM2853 P12 *ygaZH*	DM2853 with pJOE5304 P12 *ygaZH*	This study
pJOE5304.1	Expression vector with *lacI* ^*q*^‐P_*tac*_‐*eGFP*, Cm^R^	Graf and Altenbuchner (2014)
pJOE5304	pJOE5304.1 ΔlacI^q^‐P_*tac*_–*eGFP*, Cm^R^	This study
pJOE5304 P93 *metK*	pJOE5304 PM1‐93 *metK*, Cm^R^	This laboratory
pJOE5304 *metB*	pJOE5304 PM1‐93 *metB*, Cm^R^	This study, promoter (Lu et al., 2012)
pJOE5304 *grxA*	pJOE5304 PM1‐93 *grxA*, Cm^R^	This study
pJOE5304 *pspE*	pJOE5304 PM1‐93 *pspE*, Cm^R^	This study
pJOE5304 P93 *ygaZH*	pJOE5304 PM1‐93 *ygaZH*, Cm^R^	This study
pJOE5304 P12 *metB*	pJOE5304 PM1‐12 *metB*, Cm^R^	This laboratory
pJOE5304 P12 *ygaZH*	pJOE5304 PM1‐12 *ygaZH*, Cm^R^	This study, Promoter (Lu et al., 2012)
ch207	ATGCAAACCGTTATTTTTGGTCG	Amplification *grxA* from gDNA
ch208	TCAGGCGTCCAGATTTTCTTTCACC	Amplification *grxA* from gDNA
ch217	ATGTTTAAAAAAGGCTTACTTGCTCTGGC	Amplification *pspE* from gDNA
ch218	TTAACCTTTGACCTTCGGCATTGC	Amplification *pspE* from gDNA
ch225	ATGACGCGTAAACAGGCCAC	Amplification *metB* from gDNA
ch226	TTACCCCTTGTTTGCAGCCC	Amplification *metB* from gDNA
ch237	TAGCATGTACGTTTAAACCAGGAAACAGCTATGCAAACCGTTATTTTTGG	Construction of pJOE5304 *grxA*
ch238	CTTCTCTCATCCGCCAAAACAGCCAAGCTTTCAGGCGTCCAGATTTTC	Construction of pJOE5304 *grxA*
ch241	TAGCATGTACGTTTAAACCAGGAAACAGCTATGTTTAAAAAAGGCTTACTTGCTCTGGC	Construction of pJOE5304 *pspE*
ch242	CTTCTCTCATCCGCCAAAACAGCCAAGCTTTTAACCTTTGACCTTCGGCATTGC	Construction of pJOE5304 pspE
ch247	TAGCATGTACGTTTAAACCAGGAAACAGCTATGACGCGTAAACAGGCCAC	Construction of pJOE5304 *metB*
ch248	CTTCTCTCATCCGCCAAAACAGCCAAGCTTTTACCCCTTGTTTGCAGCCC	Construction of pJOE5304 metB
ch352	ATGGAAAGCCCTGCACCC	Amplification of *ygaZH*
ch353	TTATAAAATGACCTCTATCTTCCAGGCGAG	Amplification of *ygaZH*
ch354	GCGTCAGTCAGTTTAAACCAGGAAACAGCTATGGAAAGCCCTGCACCC	Construction of pJOE5304 P12 *ygaZH*
ch355	CTTCTCTCATCCGCCAAAACAGCCAAGCTTTTATAAAATGACCTCTATCTTCCAGGCGAG	Construction of pJOE5304 P12 *ygaZH* and pJOE5304 P93 *ygaZH*
ch356	TAGCATGTACGTTTAAACCAGGAAACAGCTATGGAAAGCCCTGCACCC	Construction of pJOE5304 P93 *ygaZH*
ch231	AGCTGTTTCCTGGTTTAAACG	Plasmid backbone with PM1‐93
ch25	AAGCTTGGCTGTTTTGGC	Plasmid backbone with PM1‐93 and PM1‐12
ch296	AGCTGTTTCCTGGTTTAAAC	Plasmid backbone with PM1‐12
ch375	CTCAGTAAACCTAAAACCGCCC	qPCR *ygaZ*
ch376	AGCCTTTCAGCAATCCGCTC	qPCR *ygaZ*
ch379	TGTTGCTCGACACCATTG	qPCR *ygaH*
ch380	GTCTTGTAGAAACTGACGCC	qPCR *ygaH*
ch387	TTGTCGGCGGTGGTGATGTC	qPCR *cysG*
ch388	ATGCGGTGAACTGTGGAATAAACG	qPCR *cysG*

*Note*: Underlined: complementary region to backbone.

### SDS‐PAGE

Cells were resuspended in 500 μL of sodium phosphate buffer (0.1 M, pH 7.0) at ⁓3 g/L. Cells were maintained on ice for the following steps: sonication cell lysis (amplitude 90%, up to 5 cycles at 1 min each; Bandelin, Berlin, Germany); centrifugation (20,800 g, 45 min, 4°C); separation of supernatant and pellet. The pellet was resuspended in the same volume of sodium phosphate buffer as the supernatant. The supernatant and pellet phases were each mixed with 5x SDS sample buffer (250 mM TRIS base/HCl pH 6.8, 10 mM EDTA, 5% [w/v] SDS, 50% [v/v] glycerin, 0.1% [w/v] bromophenol blue and 5% [v/v] β‐mercaptoethanol) and boiled at 95°C for 5 min. A total of 15 μL of each phase was loaded onto the gel (separating gel: 375 mM Tris base/HCl pH 8.8, 0.1% [v/v] SDS, 15% [v/v] or 18% [v/v] acrylamide/bis‐acrylamide solution [29:1], 0.05% [v/v] APS, 10 μL TEMED; stacking gel: 125 mM Tris base/HCl pH 8.8, 0.1% [v/v] SDS, 5% [v/v] acrylamide/bis‐acrylamide solution [29:1], 0.1% [v/v] APS, 5 μL TEMED). Depending on the protein size, different running buffers were used: proteins >30 kDa: 250 mM Tris base, 2.5 M glycine and 1% (w/v) SDS; proteins <30 kDa: cathode buffer (0.1 M Tris base, 0.1 M tricine and 0.1% [w/v] SDS) and anode buffer (0.1 M Tris base, pH 8.9). Electrophoresis (Bio‐Rad Laboratories, Hercules, CA, USA) was performed at 120 V for 10 min and then at 200 V for 60–110 min. This was followed by a 20 min incubation in staining solution (0.2% [w/v] Coomassie R250, 0.05% [w/v] Coomassie G250, 42.5% [v/v] ethanol, 5% [v/v] methanol and 10% [v/v] acetic acid), 20 min incubation in destaining solution (45% [v/v] ethanol and 10% [v/v] methanol) and overnight incubation in 7.5% (v/v) acetic acid.

### LC–MS analysis

Targeted and quantitative analyses of intracellular *E. coli* metabolite pools in the l‐Met synthesis network were based on previous HILIC‐ESI‐MS studies with pre‐optimized mass transitions and adapted QQQ‐MS/MS parameters (Feith et al., 2019; Frank et al., 2020; Teleki et al., 2015). Measurements were performed on HPLC (Agilent 1200 Series) coupled with an Agilent 6410 B triple quadrupole tandem mass spectrometer (QQQ‐MS/MS; Agilent Technologies, Waldbronn, Germany). System control, acquisition and data analysis were performed using commercial MassHunter B.07.00 software. Sampling and extraction were performed according to the following adapted procedure: 2 mL of biosuspension was harvested and immediately centrifuged at 12,100 × g for 20 s (MiniSpin, Eppendorf AG, Hamburg, Germany). The supernatant was discarded, and the pellet was washed with 1.5 mL isotonic 0.9% [w/v] sodium chloride solution. After a second centrifugation step (12,100*g* for 20 s), the supernatant was discarded, and the cell pellet was immediately frozen in liquid nitrogen and temporarily stored at −70°C. The frozen pellets were resuspended in pre‐cooled (−20°C) extraction buffer consisting of 50% [v/v] methanol and 150 μM l‐norvaline (internal standard). The volumes were adjusted to achieve constant biomass (cell dry weight) concentrations of 5–20 g/L. During resuspension, the sample temperature was maintained below −20°C by rotational vortexing (<1 min) and chilling in a constantly cooled cryostat (−40°C) (RK20, Lauda, Germany). The same volume of pre‐cooled chloroform (−20°C) was added to the fully resuspended pellets, incubated for 1 h at −20°C in a rotary overhead shaker and subsequently vortexed for 1 h at room temperature. The remaining cell debris was separated by centrifugation (20,000 g for 10 min at 4°C) (5804 R, Eppendorf AG, Hamburg, Germany). The upper H_2_O/methanol phase (polar metabolites) was stored at −70°C until measurement. For quantitative measurements of the reactive thiol group‐containing metabolites Cys and HCys and reduced glutathione, an adapted pre‐column derivatization was required. According to a previous protocol (Ortmayr et al., 2015), the sample extract was incubated with 20 mM N‐ethylmaleimide (NEM) and 10 mM ammonium acetate (pH 7.0) for 15 min at room temperature. The resulting NEM‐derivatives were detected using previously established precursor‐to‐product ion transitions and optimized QQQ‐MS/MS parameters. For extracellular metabolite pools, the biosuspension was centrifuged at 12,100*g* for 3 min at room temperature. Prior to LC–MS measurement, an additional filtration step was necessary using a centrifugation unit RotiSpin MINI‐3 (Carl Roth GmbH + Co. KG, Karlsruhe, Germany) at 20,000*g* for 10 min at room temperature. Samples were stored at −20°C until measurement. Generated data is used to calculate the intracellular and extracellular metabolite concentration in μM, μmol/gDCW or % μmol/gDCW. The sample calculation is presented in the Supplementary Tables (Table S1 and S2) Relative quantification of Homolanthionine (HLan) was achieved using targeted and adapted mass transitions. Selective precursor‐to‐product ion transitions ([*M* + *H*]^+^ ➔ *m*/*z* = 237 to [*M*]^+^ ➔ *m*/*z* = 56) and associated MS/MS parameters (collision energy = 27 V and fragmentor = 85 V) were previously determined by collision‐induced dissociation (CID) studies of the reference standards HCys and Cysta and validated by ^13^C labelling experiments (Teleki, 2016).

## RESULTS

### Identification of potential bottlenecks in the parental strain *E. Coli* DM2853

**FIGURE 2:**
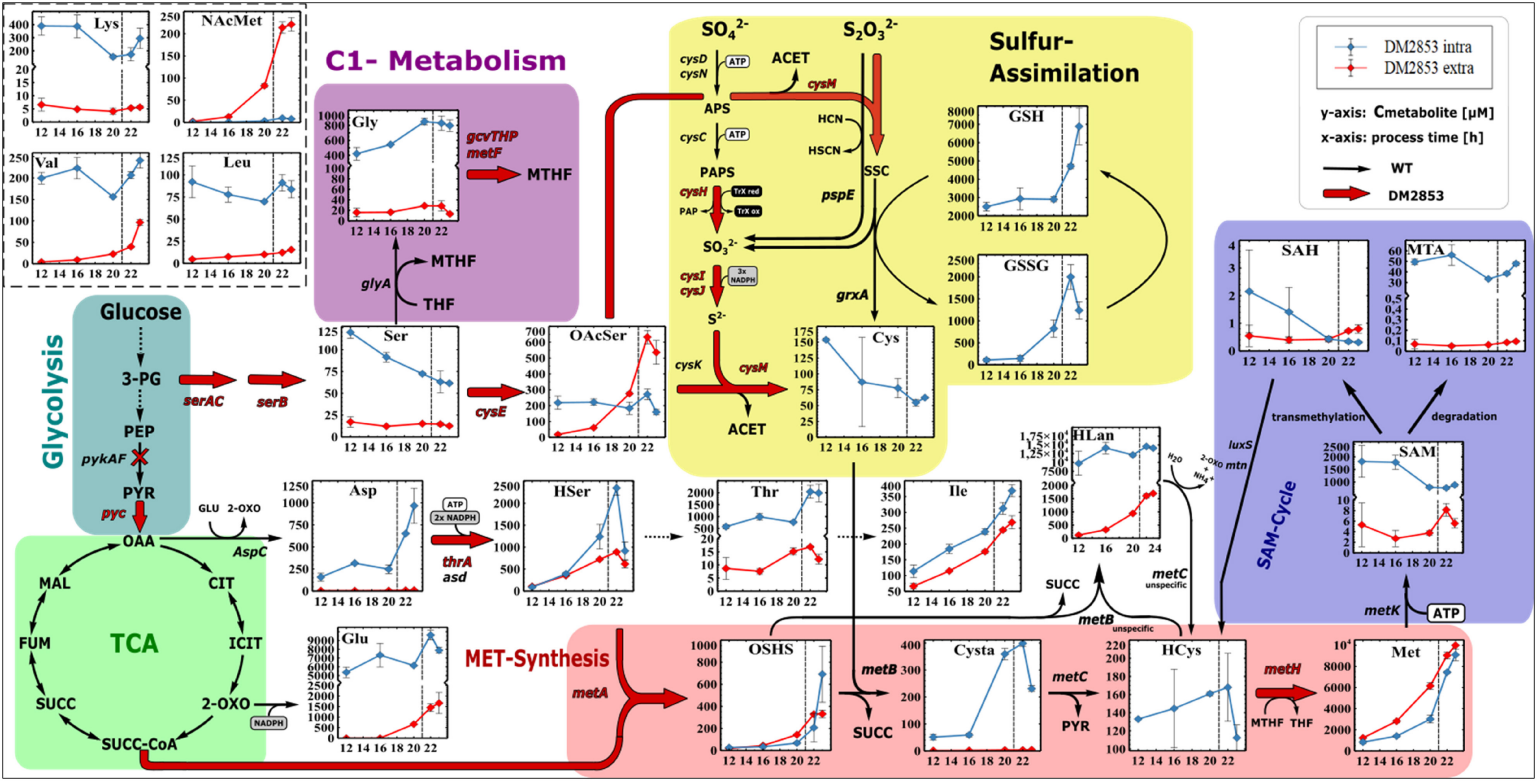
Metabolic map of the l‐methionine synthesis pathway of *E. coli* DM2853 with the intracellular and extracellular metabolite concentration during cultivation measured by LC‐MS/MS. Vertical dotted line in the graphs indicates the depletion of glucose. Black arrows depict wild type enzymatic reaction. Thick red arrows depict engineered enzymatic reaction of *E. coli* DM2853. Dotted arrow indicates more than one enzymatic reaction. Red cross indicates knockout. Values represent arithmetic mean with standard error of biological triplicates. 2‐OXO, 2‑oxoglutarate; 3‑PG, 3‑phosphoglycerate; ACET, acetate; APS, adenosine phosphosulfate; Asp, l‐aspartate; CIT, citrate; Cys, l‑cysteine; Cysta, l‐cystathionine; FUM, fumarate; Glu, l‑glutamate; Gly, l‐glycine; GSH, reduced glutathione; GSSG, oxidized glutathione; HCN, hydrogen cyanide; HCys, l‐homocysteine; HLan, l‑homolanthionine; HSCN, thiocyanate; HSer, l‐homoserine; ICIT, isocitrate; Ile, l‐isoleucine; Leu, l‐leucine; Lys, l‑lysine; MAL, malate; Met, l‐methionine; MTA, methylthioadenosine; MTHF, 5‐methyltetrahydrofolate; NAcMet, *N*‐acetyl‐l‐methionine; OAA, oxaloacetate; OAcSer, *O*‐acetyl‐l‐serine; OSHS, O‑succinyl‐l‐homoserine; PAPS, phosphoadenosine phosphosulfate; PEP, phosphoenolpyruvate; PYR, pyruvate; SAH, S‐adenosyl‐l‐homocysteine; SAM, S‐adenosyl‐l‐methionine; Ser, l‐serine; SSC, S‐sulfo‐l‐cysteine; SUCC, succinat; SUCC‐CoA, succinyl‐coenzyme A; THF, Tetrahydrofolate; Thr, l‐threonine; Val, l‐valine, pykAF encoding pyruvate kinase I+II, pyc encoding pyruvate carboxylase, metA encoding homoserine O‐succinyltransferase, metB encoding O‐succinylhomoserine lyase, metC encoding cystathionine β‐lyase, metH encoding cobalamin‐dependent methionine synthase, metK encoding methionine adenosyltransferase, aspC encoding aspartate aminotransferase, thrA encoding aspartate kinase I, asd encoding aspartate‐semialdehyde dehydrogenase, serA encoding phosphoglycerate dehydrogenase, serC encoding phosphoserine aminotransferase, serB encoding phosphoserine phosphatase, cysE encoding serine acetyltransferase, cysK encoding cysteine synthase A, cysM encoding cysteine synthase B, cysDN encoding sulfate adenylyltransferase, cysC encoding adenylyl‐sulfate kinase, cysH encoding phosphoadenosine phosphosulfate reductase, cysIJ encoding sulfite reductase, pspE encoding thiosulfate sulfurtransferase, grxA encoding reduced glutaredoxin 1, glyA encoding serine hydroxymethyltransferase, gcvT encoding aminomethyltransferase, gcvH encoding glycine cleavage system H protein, gcvP encoding glycine decarboxylase, metF encoding 5,10‐methylenetetrahydrofolate reductase.

Shake flask cultivation followed by metabolite analysis was performed to determine the potential bottlenecks in methionine production in *E. coli* DM2853. Samples were collected during the exponential growth and stationary phase. The transition is indicated by the vertical dotted line at 21 h that reflects glucose depletion. The intra‐ and extracellular metabolite pools along the synthesis pathway were quantified using LC–MS/MS (Figure 2). As the complete serine pathway (*serA*, *serC*, *serB* and *cysE*) is overexpressed, the concentrations of Ser and OAcSer were investigated. The extracellular Ser concentration was maintained, whereas the intracellular Ser concentration steadily decreased. The subsequent metabolite OAcSer exhibited the opposite behaviour. The intracellular concentration remained constant, whereas the extracellular concentration increased and exceeded the intracellular concentration. Considering an intra‐ to extracellular volume ratio of approximately 1:180, the extracellular amount exceeded the intracellular content of OAcSer by approximately 360‐fold. Apparently, the cell prefers to export OAcSer instead of metabolizing it. This indicates the potential existence of a reaction bottleneck downstream of OAcSer. There was an initial surplus of Ser production within the first 12 h, and this was followed by a steady requirement that remained until the end of the process.

Sulfur assimilation occurs downstream of OAcSer. Within this component of the metabolism, the sulfate and thiosulfate ABC transporter, *cysH*, *cysIJ* and *cysM* are overexpressed. The metabolites Cys, reduced glutathione (GSH) and oxidized glutathione (GSSG) were quantified (Figure 2). The study focused on intracellular analysis, as related extracellular amounts were deemed to be very low and mirrored the lack of alternative sulfur reduction systems outside the cells. Notably, intracellular GSH levels were found to be the second highest of all metabolites monitored in this study, and this was followed only by intracellular l‐Met content. During the exponential growth phase, the concentrations of GSH and GSSG increased but with different dynamics. As soon as the strain entered the stationary phase, the concentration of GSSG decreased, whereas that of GSH increased. As indicated in Table 2, the GSH/GSSG ratio decreased from approximately 23 to 3 during the exponential growth and increased again in the stationary phase. Given that the Cys levels downstream of GSSG remained low in the stationary phase, the coinciding decline in GSSG and increase in GSH may mimic the amplified back reaction. Interestingly, the intracellular Cys pools decreased steadily during cultivation. Concurrently, replenishment via GSSG/GSH and OAcSer should have been possible due to the very high pool sizes and observed exports, respectively. Given that GSH is an important scavenger molecule (Sies, 1999), large pool sizes may reflect cellular efforts to prepare for expected cellular stress. Alternatively, the discussion of the correlated GSH/Cys pools leads to the assumption that there is a bottleneck in the sulfur assimilation. Apparently, sulfur metabolism via *cysK*, *cysM* and *grxA* requires an optimized adjustment to improve Cys supply. Simultaneously, excessively high Cys levels are toxic to *E. coli* (Kari et al., 1971; Nagy et al., 1969; Sorensen & Pedersen, 1991). Therefore, the fluxes of sulfur assimilation must be enhanced and carefully balanced. Otherwise, the supply of Cys is likely to limit l‐Met biosynthesis or cell growth.

**TABLE 2:** GSH/GSSG – ratio in the exponential growth phase and the stationary phase of DM2853, DM2853 metB, DM2853 grxA and DM2853 pspE.

GSH/GSSG				
Growth	DM2853	metB	grxA	pspE
Exp.	23.44	30.05	8.79	1.08
	20.42	30.41	1.35	0.90
	3.53	6.22	0.67	1.03
Stat.	2.36	6.23	1.26	0.23
	5.57	12.49	1.68	0.70

Within the isoleucine (Ile) biosynthesis pathway, the intermediates Asp, homoserine (HSer), Thr and Ile were monitored (Figure 2). In this pathway, only Thr feedback‐resistant ThrA is overexpressed. Asp and Thr displayed only minor extracellular dynamics at approximately 10 μM that were equally mirrored by proportional intracellular variations. Consequently, metabolite analyses focused on HSer and Ile. Both, intra‐ and extracellular levels steadily rose, reaching levels internally of approximately 1000 μM and 300 μM for HSer and Ile, respectively, before entering the stationary phase. In particular, the steady accumulation of HSer was remarkable, as this metabolite is believed to fuel l‐Met biosynthesis. Instead, HSer and the downstream product Ile are heavily exported which indicates a metabolic bottleneck finally converts HSer into Met.

Within l‐Met biosynthesis, l‐Met and the SAM feedback‐resistant MetA (Q64E) is overexpressed (Tang, Chen, et al., 2020; Tang, Du, et al., 2020). Transcriptional repression of l‐Met synthesis (*metABC*) is lowered, as *metJ* deletion and l‐Met formation are fostered by the overexpression of *metH*. All monitored pathway intermediates (Figure 2), including OSHS, Cysta, HCys and l‐Met, exhibited increasing internal pool sizes during exponential growth, thus resembling the dynamics of the precursor HSer. Interestingly, the internal pool level of l‐Met exceeded that of the others by approximately one order of magnitude. Given that extracellular l‐Met levels are higher than intracellular concentrations, active export should have occurred, albeit not sufficiently strongly enough to prevent internal l‐Met accumulation.

The (asymmetric) thioether Cysta is synthesized by the conversion of OSHS and Cys using MetB. Previous studies (Krömer et al., 2006; Teleki, 2016) have demonstrated that increasing intracellular levels of cytotoxic HCys, which can be observed in DM2853 (Figure 2), leads to an additional non‐specific conversion of OSHS and HCys to the symmetric thioether Homolanthionine (HLan). Subsequently, MetC non‐specifically converts HLan to HCys and 2‐oxoglutarate (2‐OXO) (Teleki, 2016). As a reference standard, HLan is not commercially available, and it was relatively quantified using the adapted mass transitions and MS/MS parameters. A direct comparison of relative Cysta levels with transferable analytical responsivity revealed a relatively high amount of HLan, and this is mirrored by the estimated intra‐ and extracellular pool dynamics.

Regarding by‐products, glutamate (Glu) formation was similar to l‐Met production in DM2853 (Figure 2). Other amino acids were detected only at low levels in the supernatant. Surprisingly, increasing levels of *N*‐acetyl‐l‐methionine (NAcMet) were detected externally, although methionine *N*‐acetyltransferase (encoded by *yncA*) that catalyses the reaction of l‐Met and acetyl‐CoA to NAcMet and coenzyme A is deleted.

The metabolic status quo of the parental strain yielded HLan, Glu, NAcMet and Ile as side products. Possible bottlenecks were identified in sulfur assimilation and downstream of OSHS. To overcome these limitations, a rationally designed overexpression strategy was developed. *E. coli* can use sulfate or thiosulfate for assimilation (Kawano et al., 2018). Thiosulfate may be CysM‐dependent (Nakatani et al., 2012) or ‐independent (Kawano et al., 2017). Both pathways are energetically less demanding than the sulfate pathway (Kawano et al., 2018). Hence, the overexpression strategy investigated the following scenarios. Regarding CysM‐independent amplification, *pspE* was overexpressed, whereas the CysM‐dependent scenario studied the overexpression of *grxA*. PspE performs 85% of the thiosulfate sulfurtransferase activity in *E. coli* (Cheng et al., 2008), and GrxA is one of the most effective cytoplasmic disulfide‐reducing proteins (Stewart et al., 1998). To resolve the bottleneck downstream of OSHS, *metB* was overexpressed.

### Metabolic patterns of the engineered strains DM2853 *pspE* , DM2583 *grxA* and DM2853 *metB*

**FIGURE 3:**
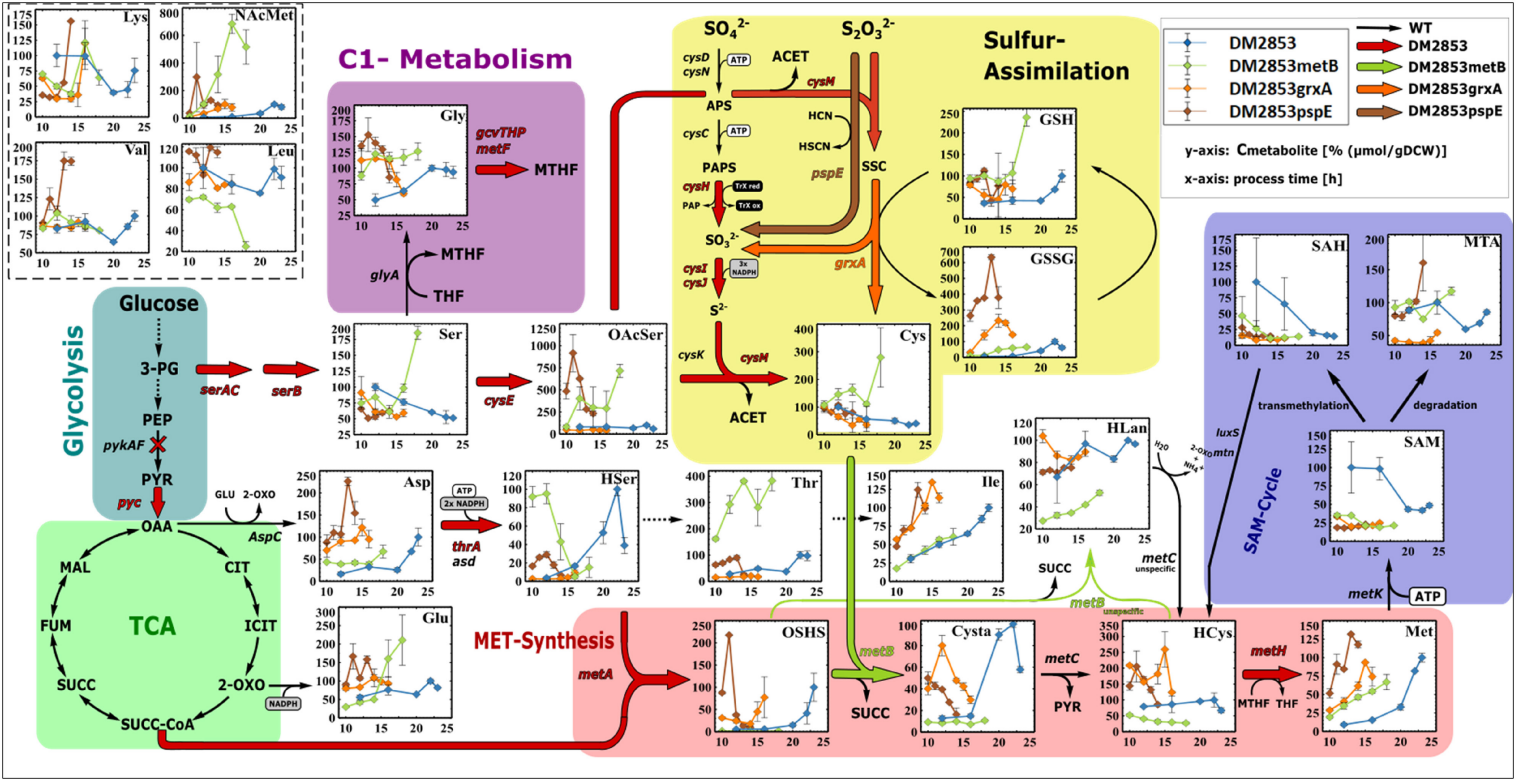
Metabolic map of the l‐methionine synthesis pathway of *E. coli* DM2853, DM2853 metB, DM2853 grxA and DM2853 pspE with the intracellular metabolite concentration during cultivation measured by LC‐MS/MS. The last two data points in every curve represent the stationary phase. Black arrows depict wild type enzymatic reaction. Thick colored arrows depict overexpressed enzymatic reactions of the engineered *E. coli* strains. Dotted arrow indicates more than one enzymatic reaction. Red cross indicates knockout. Values represent arithmetic mean with standard error of biological triplicates. Lys: L‑lysine, NAcMet: N‐acetyl‐L‐methionine, Val: L‐valine, Leu: L‐leucine, Gly: L‐glycine, Ser: L‐serine, OAcSer: O‐acetyl‐L‐serine, Cys: L‑cysteine, GSH: reduced glutathione, GSSG: oxidized glutathione, Asp: L‐aspartate, HSer: L‐homoserine, Thr: L‐threonine, Ile: L‐isoleucine, HLan: L‑homolanthionine, Glu: L‑glutamate, OSHS: O‑succinyl‐L‐homoserine, Cysta: L‐cystathionine, HCys: L‐homocysteine, Met: L‐methionine, SAM: S‐adenosyl‐L‐methionine, MTA: methylthioadenosine, SAH: S‐adenosyl‐L‐ homocysteine, 3‑PG: 3‑phosphoglycerate, PEP: phosphoenolpyruvate, PYR: pyruvate, OAA: oxaloacetate, CIT: citrate, ICIT: isocitrate, 2‐OXO: 2‑oxoglutarate, SUCC‐CoA: succinyl‐coenzyme A, SUCC: succinate, FUM: fumarate, MAL: malate, MTHF: 5‐methyltetrahydrofolate, THF: Tetrahydrofolate, APS: adenosine phosphosulfate, PAPS: phosphoadenosine phosphosulfate, SSC: S‐sulfo‐L‐cysteine, HCN: hydrogen cyanide, HSCN: thiocyanate, ACET: acetate. pykAF encoding pyruvate kinase I+II, pyc encoding pyruvate carboxylase, metA encoding homoserine O‐succinyltransferase, metB encoding O‐succinylhomoserine lyase, metC encoding cystathionine β‐lyase, metH encoding cobalamin‐dependent methionine synthase, metK encoding methionine adenosyltransferase, aspC encoding aspartate aminotransferase, thrA encoding aspartate kinase I, asd encoding aspartate‐semialdehyde dehydrogenase, serA encoding phosphoglycerate dehydrogenase, serC encoding phosphoserine aminotransferase, serB encoding phosphoserine phosphatase, cysE encoding serine acetyltransferase, cysK encoding cysteine synthase A, cysM encoding cysteine synthase B, cysDN encoding sulfate adenylyltransferase, cysC encoding adenylyl‐sulfate kinase, cysH encoding phosphoadenosine phosphosulfate reductase, cysIJ encoding sulfite reductase, pspE encoding thiosulfate sulfurtransferase, grxA encoding reduced glutaredoxin 1, glyA encoding serine hydroxymethyltransferase, gcvT encoding aminomethyltransferase, gcvH encoding glycine cleavage system H protein, gcvP encoding glycine decarboxylase, metF encoding 5,10‐methylenetetrahydrofolate reductase.

**FIGURE 4:**
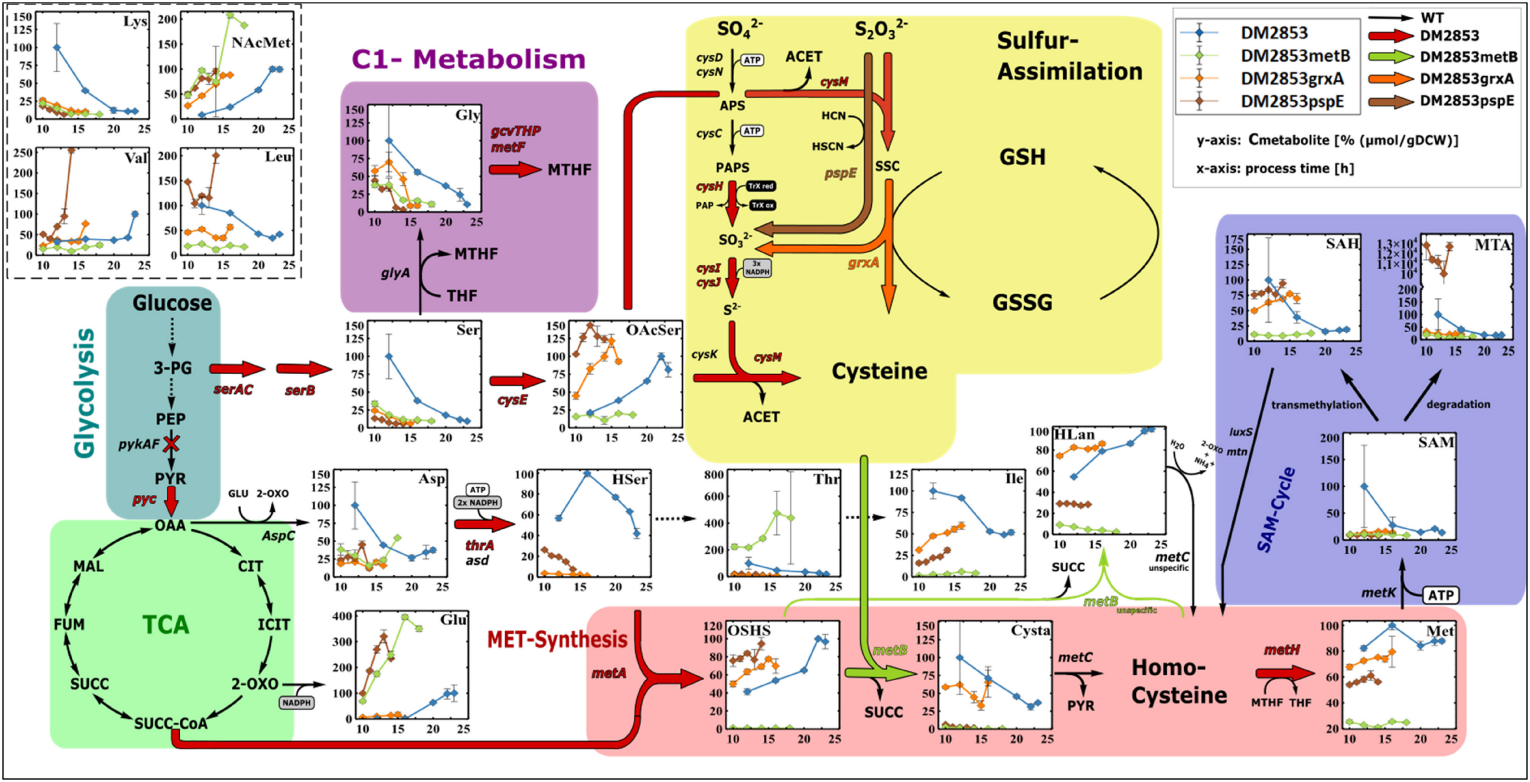
Metabolic map of the L‐methionine synthesis pathway of *E. coli* DM2853, DM2853 metB, DM2853 grxA and DM2853 pspE with the extracellular metabolite concentration during cultivation measured by LC‐MS/MS. The last two data points in every curve represent the stationary phase. Black arrows depict wild type enzymatic reaction. Thick colored arrows depict overexpressed enzymatic reactions of the engineered *E. coli* strains. Dotted arrow indicates more than one enzymatic reaction. Red cross indicates knockout. Values represent arithmetic mean with standard error of biological triplicates. 2‐OXO, 2‑oxoglutarate; 3‑PG, 3‑phosphoglycerate; ACET, acetate; APS, adenosine phosphosulfate; Asp, l‐aspartate; CIT, citrate; Cys, l‑cysteine; Cysta, l‐cystathionine; FUM, fumarate; Glu, l‑glutamate; Gly, l‐glycine; GSH, reduced glutathione; GSSG, oxidized glutathione; HCN, hydrogen cyanide; HCys, l‐homocysteine; HLan, l‑homolanthionine; HSCN, thiocyanate; HSer, l‐homoserine; ICIT, isocitrate; Ile, l‐isoleucine; Leu, l‐leucine; Lys, l‑lysine; MAL, malate; Met, l‐methionine; MTA, methylthioadenosine; MTHF, 5‐methyltetrahydrofolate; NAcMet, *N*‐acetyl‐l‐methionine; OAA, oxaloacetate; OAcSer, O‐acetyl‐l‐serine; OSHS, O‑succinyl‐l‐homoserine; PAPS, phosphoadenosine phosphosulfate; PEP, phosphoenolpyruvate; PYR, pyruvate; SAH, *S*‐adenosyl‐l‐homocysteine; SAM, S‐adenosyl‐l‐methionine; Ser, l‐serine; SSC, S‐sulfo‐l‐cysteine; SUCC, succinate; SUCC‐CoA, succinyl‐coenzyme A; THF, Tetrahydrofolate; Thr, l‐threonine; Val, l‐valine; pykAF encoding pyruvate kinase I+II, pyc encoding pyruvate carboxylase, metA encoding homoserine O‐succinyltransferase, metB encoding O‐succinylhomoserine lyase, metC encoding cystathionine β‐lyase, metH encoding cobalamin‐dependent methionine synthase, metK encoding methionine adenosyltransferase, aspC encoding aspartate aminotransferase, thrA encoding aspartate kinase I, asd encoding aspartate‐semialdehyde dehydrogenase, serA encoding phosphoglycerate dehydrogenase, serC encoding phosphoserine aminotransferase, serB encoding phosphoserine phosphatase, cysE encoding serine acetyltransferase, cysK encoding cysteine synthase A, cysM encoding cysteine synthase B, cysDN encoding sulfate adenylyltransferase, cysC encoding adenylyl‐sulfate kinase, cysH encoding phosphoadenosine phosphosulfate reductase, cysIJ encoding sulfite reductase, pspE encoding thiosulfate sulfurtransferase, grxA encoding reduced glutaredoxin 1, glyA encoding serine hydroxymethyltransferase, gcvT encoding aminomethyltransferase, gcvH encoding glycine cleavage system H protein, gcvP encoding glycine decarboxylase, metF encoding 5,10‐methylenetetrahydrofolate reductase.

Plasmid‐based overexpression of *pspE*, *grxA*, and *metB* resulted in the strains DM2853 *pspE*, DM2853 *grxA*, and DM2853 *metB*. Overexpression was confirmed by SDS‐PAGE (Figure S1). All strains exhibited faster growth than that of the parental strain (Figure S3). The metabolic conditions of these strains were analysed by LC–MS/MS. The intracellular and extracellular concentrations are presented in Figures 3 and 4, respectively.

Regarding GSH and GSSG (Figure 3), all engineered strains displayed higher levels of GSH and GSSG than that of DM2853. After entering the stationary phase, GSSG levels decreased. The GSH/GSSG ratios exhibited similar dynamics in all strains, although increased GSSG levels significantly reduced the ratios after *grxA* and *pspE* overexpression (Table 2). Apparently, overexpression of these genes improved electron transfer for reducing thiosulfate, ultimately fuelling the supply of SO_3_^2−^ for l‐Met biosynthesis. However, increasing pool sizes of the downstream intermediate Cys were observed only in DM2853 *metB*. Intracellular pool dynamics in DM2853 *grxA* and DM2853 *pspE* did not change significantly compared to that of the parental strain.

To engineer a potential bottleneck downstream of OSHS, *metB* was overexpressed (Figures 3 and 4). MetB catalyses the conversion of OSHS and Cys to Cysta and succinate (Holbrook et al., 1990). MetB also converts OSHS and HCys into HLan and 2‐OXO with promising activities (Teleki, 2016). Surprisingly, HLan levels decreased after *metB* overexpression in DM2853 *metB* cells, whereas they persisted in all the other strains. Likewise, *metB* overexpression reduced the levels of the downstream intermediates Cysta and HCys, whereas the overexpression of *grxA* and *pspE* resulted in larger pool sizes compared to the parental strain (Figure 3). In all cases, the intracellular concentrations of the targeted product l‐Met increased significantly, thus indicating the strongest dynamic after *pspE* overexpression (Figure 3). However, extracellular titers of l‐Met (Figure 4) were the highest for the parental strain, and this was followed by those of the engineered *grxA*, *pspE* and *metB* strains. The latter reflects the surprisingly downregulated activity that has already been observed in intracellular pools. Comparing the intra‐ and extracellular l‐Met levels, much faster intracellular accumulation (Figure 3) was observed in the *grxA* and *pspE* amplified strains, with the latter exhibiting the fastest dynamics.

Most of the extracellular amino acid levels (Figure 4) decreased in the engineered strains compared to those in DM2853. The exceptions were external Thr, Glu and NAcMet levels in DM2853 *metB* and Glu, valine (Val) and leucine (Leu) in the supernatant of DM2853 *pspE*. Given that the overexpression of *metB* unexpectedly caused the downregulation of l‐Met synthesis (also mirrored by the reduced side product HLan), the accumulation of external Thr and Glu may reflect backlogs of carbon flux branching off from the TCA precursors oxaloacetate (OAA) and 2‐OXO. NAcMet does not reflect reduced l‐Met synthesis, as only ~6% of the lost l‐Met can be observed in NAcMet. By analogy, the occurrence of the branched‐chain amino acids Val and Leu may mirror detouring from the precursor pyruvate.

### Impact of the Met exporter

**FIGURE 5:**
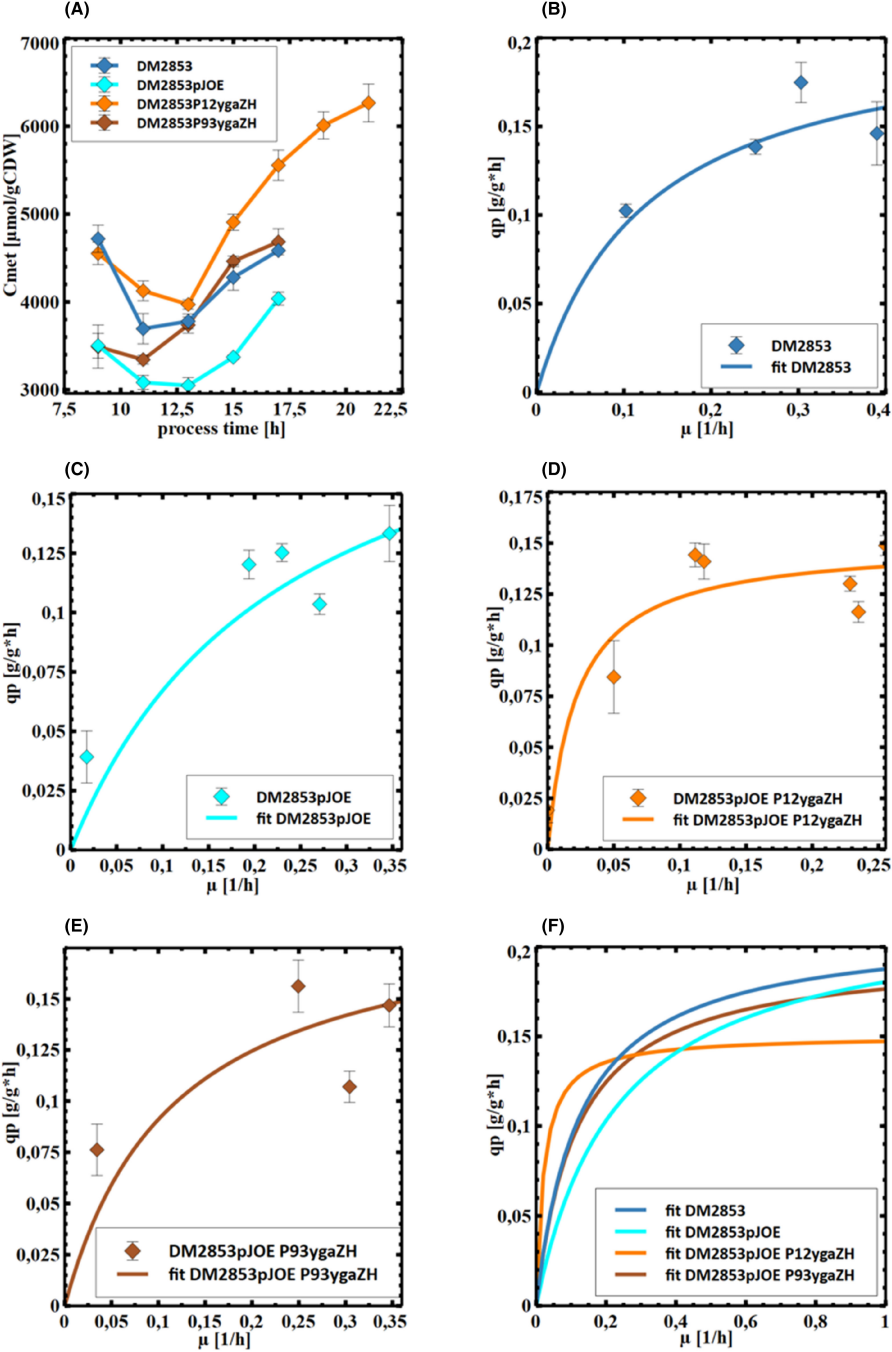
Influence of the overexpressed exporter YgaZH on the (A) biomass‐specific methionine concentration. (B–E) methionine export rate and line of best fit. (F) Overlay of all lines of best fit from B to E. Values represent arithmetic mean with standard error of biological triplicates.

Considering the rising intracellular l‐Met levels, even in the parental strain DM2853 (Figure 3), studies aiming to overexpress Met export were performed. The branched‐chain amino acid exporter YgaZH mediates the export of l‐Val and l‐Met (Figge et al., 2016). Overexpression of *ygaZH* leads to improved l‐Met production in *E. coli* (Park et al., 2008). Therefore, this exporter was overexpressed when high and moderate promoter strengths were used in DM2853 P93 *ygaZH* and DM2853 P12 *ygaZH*, respectively (see qPCR in figure S2 for documentation and the growth curves in Figure S4). However, the glucose‐to‐l‐Met yields remained unchanged (Table 3), and the analysis of l‐Met per biomass revealed the superior performance of DM2853 P12 *ygaZH* (Figure 5A). Further studies examining the biomass‐specific l‐Met formation rates (*q*_*p*_) as a function of growth (μ, Figure 5B–E) disclosed asymptotic maximum product formation that could be modelled via Michaelis–Menten type saturation kinetics. Figure 5F indicates that DM2853, DM2853 pJOE and DM2853 P93 *ygaZH* maintained similar *q*_*p*_ kinetics, whereas DM2853 P12 *ygaZH* reached maximum *q*_*p*_ already at lower growth rates at the expense of lower maximum *q*_*p*_ values.

**TABLE 3:** Influence of the overexpressed exporter YgaZH on the methionine from glucose yield (*Y*_Met/S_).

	*Y*_Met/S_ [%, g/g]	SD
DM2853	12.02	0.09
DM2853pJOE	13.19	0.32
DM2853pJOE P12ygaZH	13.82	1.05
DM2853pJOE P93ygaZH	13.83	0.29

*Note*: Values represent arithmetic mean with standard deviation of biological triplicates.

## DISCUSSION

The l‐Met producing strain *E. coli* DM2853 was successfully developed by a previous research study (Dischert & Figge, 2013). As indicated in Table 3, the strain is well able to produce l‐Met from glucose with a considerable conversion yield of 12%–13% (g/g) that was 14–16 mol %. Specifically, approximately 12%–13% of the consumed glucose is drained into the biosynthesis of l‐Met, and this is approximately 26.6‐fold greater than the cellular needs of a wild‐type *E. coli*. The biosynthesis of l‐Met imposes a high metabolic burden on microbes. To balance the metabolic demands of the precursors OAA and succinyl‐CoA, 7 ATP and 8 NADPH are required in addition to one methyl group that must be provided via MTHF from l‐Ser. Sulfate, a common sulfur source in the media, must be reduced (ATP‐ and NADPH‐dependent) to sulfite to create l‐Cys. Hence, the challenge for l‐Met production is the need for an orchestrated improvement in the supply of all metabolic precursors, methyl groups, anabolic redox factors and energy via ATP. This is a formidable task, considering that *E. coli* aims to fine‐tune the flux into l‐Met synthesis via the central transcriptional repressor MetJ. Therefore, the microbe prevents unnecessary futile cycling while preserving a sufficient supply of the essential sulfur‐containing amino acids together with the downstream product SAM, the universal methyl group donor inside the cells.

Given that the regulator *metJ* is deleted in the *E. coli* constructs used in this study, particular focus was placed on the potential impact on metabolites in this transcriptionally deregulated scenario. Notably, all observations were performed in l‐Met‐producing cells that already possessed a significantly perturbed metabolic flux pattern compared to that of the non‐producing wild type.

One surprising finding was the result of *metB* overexpression. Considering that HLan, Glu, NAcMet and Ile were observed as side products in the reference strain, the overexpression of *metB* appears to reduce byproduct formation while strengthening l‐Met production. However, overexpression of *metB* led to unexpected results, where the concentrations of all downstream metabolites, including Met, decreased significantly. Interestingly, comparable phenomena were also observed in another *E. coli* strain, thus revealing a genetic similarity to DM2853. Overexpression of *metB* and *metL* led to reduced Met concentrations, whereas the levels of Thr and HSer increased (Huang et al., 2018). In DM2853 *metB*, intracellular Thr levels also increased remarkably and exceeded all pool sizes in other tests. Therefore, it can be hypothesized that large Thr pool sizes may impose inhibitory effects on l‐Met biosynthesis. There may also be a link between *metB* overexpression and increased Thr that is not yet understood mechanistically.

An attempt to improve sulfur assimilation by overexpressing *grxA* and *pspE* resulted in increased intracellular pool sizes for all metabolites downstream of *metB*. In particular, the accumulation of intracellular l‐Met was accelerated. Notably, the intracellular Thr pools remained low, whereas flux via MetB was enhanced. This observation may serve as a basis for the not yet elucidated interaction between MetB and Thr.

The time‐courses presented in Figure 5, particularly Figure 5F, disclose another finding. l‐Met export exhibits the typical features of an active export mechanism, thus revealing a characteristic saturation curve. Furthermore, the overexpression of the exporter *ygaZH* did not improve maximum transport capacities but instead improved the affinity for the substrate l‐Met in the Michaelis–Menten type reaction. Specifically, overexpression of *ygaZH* achieved maximum saturated export rates already at lower growth rates than those in the non‐amplified parental strain. This is a promising finding, as it opens the door for an optimized industrial process. The ideal industry‐producing strain exhibits high product formation rates even under limited growth conditions, meaning at low growth rates, to meet the technical limitations in large‐scale production. In the case of methionine synthesis, the engineered export presented here enables precise characterization of these characteristics. This is a step towards decoupling growth from production, as l‐Met biosynthesis does not have to compete with biomass formation.

## AUTHOR CONTRIBUTIONS

**Claudia Harting:** Conceptualization, Investigation, Genetic Engineering, Metabolomic experiments, Methodology, Formal Analysis, Validation, Visualization, Writing – Original Draft Preparation; writing – review and editing. **Attila Teleki:** Metabolomic experiments. **Marius Braakmann:** Formal Analysis. **Frank Jankowitsch:** Writing – review and editing, Project Administration, resources. **Ralf Takors:** Writing – Review and Editing; Funding acquisition; Project administration; resources; supervision.

## CONFLICT OF INTEREST STATEMENT

The authors declare no conflict of interest.

## Supporting information


**Data S1:** Supporting Information.
