# Supplementary material for: Systemic intracellular analysis for balancing complex biosynthesis in a transcriptionally deregulated Escherichia coli l‐Methionine producer
Source: Microb Biotechnol. 2024 Mar 25;17(3):e14433. doi: 10.1111/1751-7915.14433 (PMC10963904; doi:10.1111/1751-7915.14433)
Supplement: Supplementary file 1 — Data S1: Supporting Information. [file MBT2-17-e14433-s001.docx]

**Supplementary**


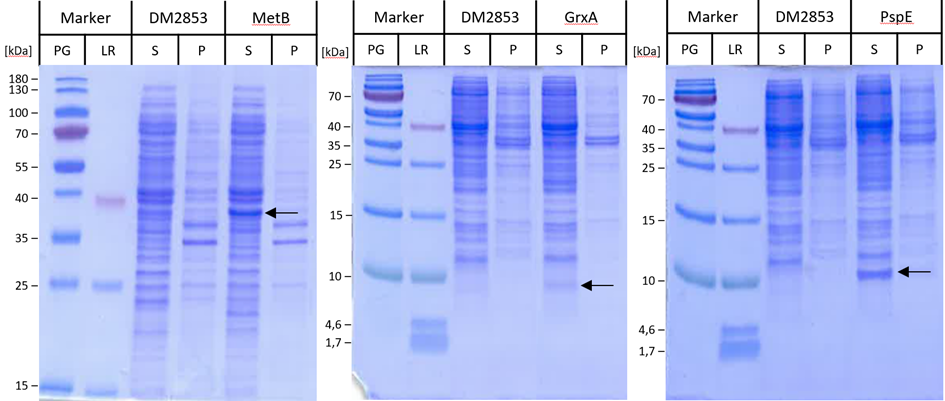


Figure S1 SDS PAGE of DM2853, DM2853 metB, DM2853 grxA and DM2853 pspE to proof overexpression of MetB (40kDa), GrxA (9kDa) and PspE (11kDa). S: soluble fraction, P: pellet fraction, PG: X2 pageruler prestained protein ladder, LR: Spectra multicolor low range protein ladder, sample gel 5%, separation gel 15 % (MetB) and 18 % (GrxA, PspE)


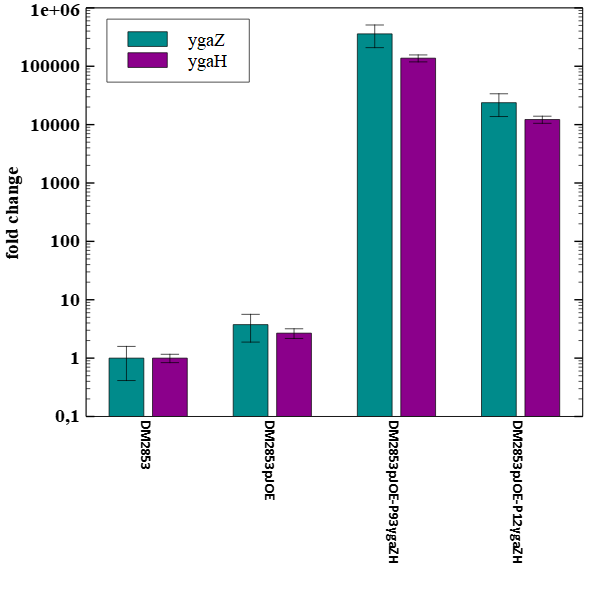


Figure S2 Evidence of overexpression of ygaZH by qPCR. pJOE: empty vector (Graf & Altenbuchner, 2014)


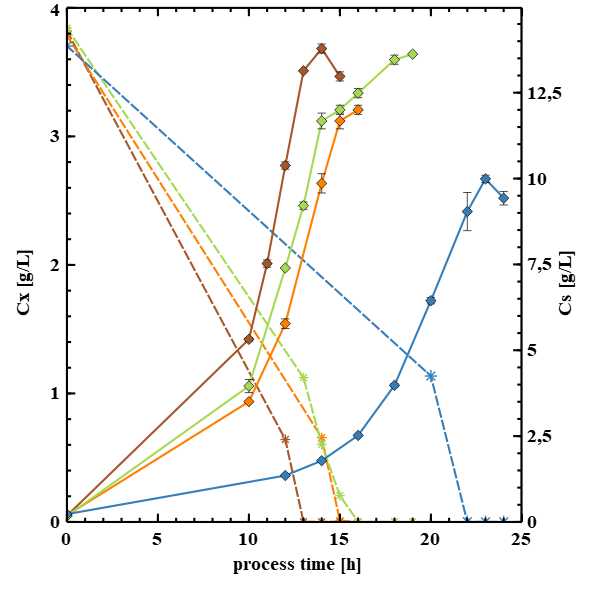


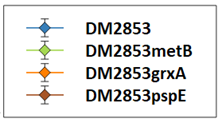


Figure S3 Growth curve (solid line) and glucose concentration (dashed line) of DM2853, DM2853metB, DM2853grxA and DM2853pspE.


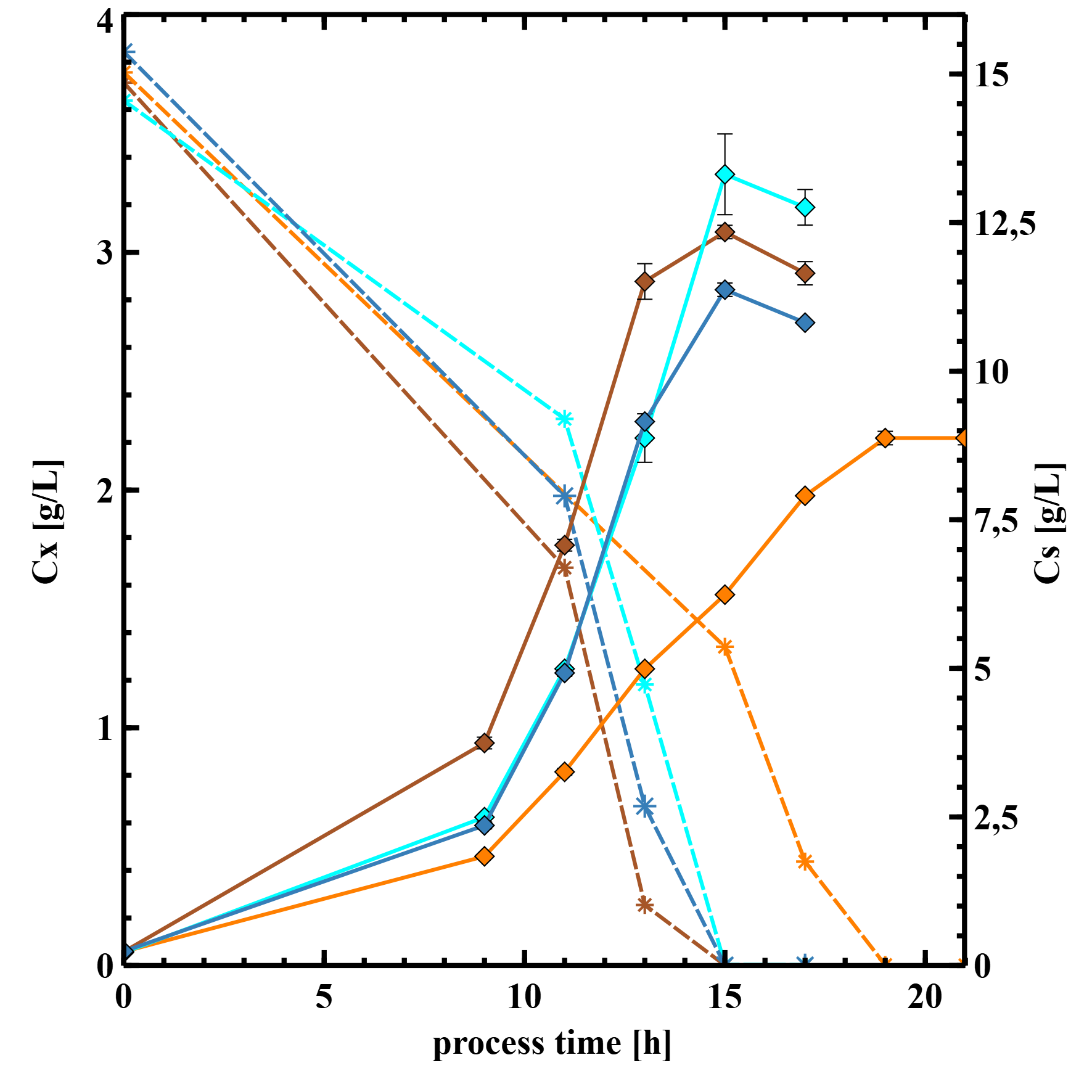


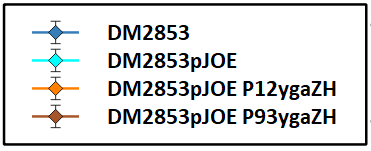


Figure S4 Growth curve (solid line) and glucose concentration (dashed line) of DM2853, DM2853pJOE, DM2853pJOE P12ygaZH and DM2853pJOE P93ygaZH

Table S1 Data and sample calculation of extracellular methionine concentration

|  | shake flask | process time [h] | DCW [g/l] | $c_{Met}$ [µM] | $c_{Met,x}$ [µmol/gDCW] |
| --- | --- | --- | --- | --- | --- |
| DM2853 | 1 | 12 | 0,36 | 1329,53 | 3652,56 |
|  |  | 16 | 0,68 | 2991,94 | 4425,95 |
|  |  | 20 | 1,77 | 6878,31 | 3890,45 |
|  |  | 22 | 2,70 | 9753,50 | 3607,07 |
|  |  | 23 | 2,70 | 9912,83 | 3665,99 |
|  | 2 | 12 | 0,36 | 1259,68 | 3460,67 |
|  |  | 16 | 0,68 | 2947,14 | 4359,67 |
|  |  | 20 | 1,66 | 5615,06 | 3374,43 |
|  |  | 22 | 2,08 | 8381,58 | 4029,60 |
|  |  | 23 | 2,60 | 10028,07 | 3856,95 |
|  | 3 | 12 | 0,34 | 1109,58 | 3282,79 |
|  |  | 16 | 0,65 | 2501,92 | 3849,10 |
|  |  | 20 | 1,77 | 5961,18 | 3371,71 |
|  |  | 22 | 2,60 | 8990,36 | 3457,83 |
|  |  | 23 | 2,81 | 10039,47 | 3575,31 |

Table S2 Data and sample calculation of extracellular methionine concentration – mean values

|  | shake flask | process time [h] | $\bar{c}_{Met}$  [µM] | $\bar{c}_{Met,x}$  [µmol/gDCW] | $\bar{c}_{Met,x,rel}$  [% µmol/gDCW] |
| --- | --- | --- | --- | --- | --- |
| DM2853 | 1+2+3 | 12 | 1232,93 | 3465,34 | 82,28 |
|  |  | 16 | 2813,67 | 4211,57 | 100,00 |
|  |  | 20 | 6151,51 | 3545,53 | 84,19 |
|  |  | 22 | 9041,81 | 3698,17 | 87,81 |
|  |  | 23 | 9993,49 | 3699,42 | 87,84 |

The following procedure showcases the calculation of the biomass specific, relative L-Methionine concentration $\bar{c}_{Met,x,rel}$ in $\% \frac{\mu mol}{gDCW}$ at 16 h. Notably, all L-Met concentrations are referenced to the related dry cell weight (DCW) leading to $c_{Met,x}$. Next, average values are calculated and the maximum value of a series is identified. All remaining values of the course are relativized to this maximum number.

The averaged, maximum concentration of L-Methionine $\bar{c}_{Met,x,max}$ was found at 16 h and is calculated as follows:

$$c_{Met,x,1}\left( 16h \right)=\frac{2991,94 \mu mol}{0,68 g/L}=4,211.57 \frac{\mu mol}{g_{DCW}}$$

$$c_{Met,x,2}\left( 16h \right)=\frac{2,947.14 \mu mol}{0,68 g/L}=4,359.67 \frac{\mu mol}{g_{DCW}}$$

$$c_{Met,x,3}\left( 16h \right)=\frac{2,501.92 \mu mol}{0,65 g/L}=3,849.10 \frac{\mu mol}{g_{DCW}}$$

$\bar{c}_{Met,x,max}\left( 16h \right)= \frac{4425,95+4359,67+3849,10}{3}=4,211.57 \frac{\mu mol}{gDCW}=100 \%$

Thereof, relative values are calculated. For example, the 12 *h* value with

$$\bar{c}_{Met,x}\left( 12h \right)= 3,465.34 \frac{\mu mol}{g_{DCW}}$$

is transferred to

$$\bar{c}_{Met,x,rel}\left( 12h \right)=\frac{3,465.34}{4,211.57}=82.28 \%\frac{\mu mol}{g_{DCW}}$$

Sequence *ygaZH* from Citrobacter koseri

ATGGAAAGCCCTGCACCCCAGTCTGAGCCCCGTCCGGCAACATTAACGGAAGGATTCAAAGACAGTTTACCGATAGTCATAAGTTATATTCCGGTGGCGTTTGCGTTTGGCCTTAACGCCACCCGTCTGGGCTTTACTCCCCTCGAAAGCGTTTTTTTCTCCTGCATTATTTACGCAGGCGCCAGCCAGTTCGTCATCACCACCATGCTCGCGGCGGGCAGCACATTATGGGTCGCCGCGCTGACCGTGATGGCGATGGACGTGCGTCATGTGCTGTACGGCCCTTCCCTGCGTAGTCGCATCAGCCAACGGCTCAGTAAACCTAAAACCGCCCTGTGGGCATTTGGCCTCACCGATGAAGTGTTTGCTGCCGCCACGGCCAAACTGGTGCGGGATAACCGCCGCTGGAGTGAAAACTGGATGATCGGCATCGCGTTCTGCTCCTGGGCCTCCTGGGTGCTCGGCACGGTCATTGGCGCATTTTCCGGGAGCGGATTGCTGAAAGGCTTCCCCGCCGTTGAGGCGGCATTAGGTTTTATGCTGCCAGCCCTGTTTATGAGCTTTTTGCTCGCTTCTTTTCAACGCAAACAAACGCTGTGCGTCACGGCGGCGTTAATCGGCGCGCTGGCAGGCGTCACGCTGTTTTCCATTCCTGCGGCTATCCTGGCGGGTATCGCCAGCGGGTGTCTGACCGCCTTGATCCAGTCGTTCTGGCAAGGAGCGCCCGATGAGTTATGAGGTTCTGCTGCTGGGACTGCTGGTCGGCTGCGCCAATTATTGTTTTCGTTATTTACCGCTTCGTCTGCGAATGGGAAACACCCGCCCCGCCAGGCGCGGCGCAACGGGCGTGTTGCTCGACACCATTGGCATCGCGTCCATCTGCGCCCTGCTGGTGGTGTCTACGGCTCCCGAAGTGATGCACGACGCCAGCCGGTTCATTCCGACGCTGGTCGGGTTTGCCGTCCTGGGCGTCAGTTTCTACAAGACGCGCAGCATCATCATCCCAACGCTACTGAGCGCTCTGGGCTATGGACTCGCCTGGAAGATAGAGGTCATTTTATAA
